# Metformin strengthens uroepithelial immunity against *E. coli* infection

**DOI:** 10.1038/s41598-021-98223-1

**Published:** 2021-09-28

**Authors:** Rakesh Kumar Majhi, Soumitra Mohanty, Witchuda Kamolvit, John Kerr White, Andrea Scheffschick, Hanna Brauner, Annelie Brauner

**Affiliations:** 1 Department of Microbiology, Tumor and Cell Biology, Karolinska Institutet, Stockholm, Sweden; 2 Division of Clinical Microbiology, Karolinska University Hospital, Stockholm, Sweden; 3 Department of Medicine, Karolinska Institutet, Stockholm, Sweden; 4 Dermatology and Venereology Clinic, Karolinska University Hospital, Stockholm, Sweden; 5 Division of Clinical Microbiology, Department of Microbiology, Tumor and Cell Biology, Karolinska Institutet and Karolinska University Hospital, 17176 Stockholm, Sweden

**Keywords:** Immunology, Microbiology, Diseases, Medical research

## Abstract

Urinary tract infection frequently caused by *E. coli* is one of the most common bacterial infections. Increasing antibiotic resistance jeopardizes successful treatment and alternative treatment strategies are therefore mandatory. Metformin, an oral antidiabetic drug, has been shown to activate macrophages in the protection against certain infecting microorganisms. Since epithelial cells often form the first line of defense, we here investigated the effect on uroepithelial cells during *E. coli* infection. Metformin upregulated the human antimicrobial peptides cathelicidin LL-37 and RNase7 via modulation of the TRPA1 channel and AMPK pathway. Interestingly, metformin stimulation enriched both LL-37 and TRPA1 in lysosomes. In addition, metformin specifically increased nitric oxide and mitochondrial, but not cytosolic ROS. Moreover, metformin also triggered mRNA expression of the proinflammatory cytokines *IL1B, CXCL8* and growth factor *GDF15* in human uroepithelial cells. The GDF15 peptide stimulated macrophages increased LL-37 expression, with increased bacterial killing. In conclusion, metformin stimulation strengthened the innate immunity of uroepithelial cells inducing enhanced extracellular and intracellular bacterial killing suggesting a favorable role of metformin in the host defense.

## Introduction

Bladder epithelial cells form the first line of defense against invading pathogens and deploy multiple intrinsic mechanisms to clear invading bacteria during urinary tract infection^1^. Innate immune molecules like antimicrobial peptides (AMPs), free radicals and cytokines contribute in controlling bacterial infection. Several AMPs, like LL-37^2–4^, and RNase7^5,6^ play a major role protecting the urinary tract against extracellular and intracellular pathogens. Therefore, strengthening the host immunity by modulatory drugs can be an alternative approach to fight infections. In the quest for such immune stimulatory compounds, we have previously demonstrated improvement of uroepithelial cell immunity by estrogen^4^, vitamin D^7^, simvastatin^8^, and traditional herbal extracts^9^. Here, we have explored the possibility of repurposing metformin (*N*,*N*-dimethylbiguanide), a frequently used anti-diabetic drug with glucose lowering effects^10^ as a therapeutic option against uropathogenic *E. coli*.

Metformin has been used clinically for several decades, with minimal side-effects even in non-diabetic patients^11,12^. Recently, metformin was shown to be effective against *Mycobacterium tuberculosis* and *Legionella pneumonia*, by inducing mitochondrial ROS production in macrophages^13,14^. This has led to the possible use of metformin complementing antibacterial drugs by stimulating host cells^15^. In this work we assessed the efficacy of metformin in strengthening the uroepithelial cells against *E. coli* infection and explored the role of the nociceptive cationic channel transient receptor potential ankyrin 1 (TRPA1) and 5' adenosine monophosphate-activated protein kinase (AMPK) pathway in metformin stimulated expression of the antimicrobial peptides LL-37 and RNase7, respectively. Our data show that metformin treatment stimulates multiple host-protective responses, resulting in increased intra- and extracellular *E. coli* killing.

## Results

### Metformin enhances the expression of the antimicrobial peptides LL-37 and RNase7

**Figure 1:**
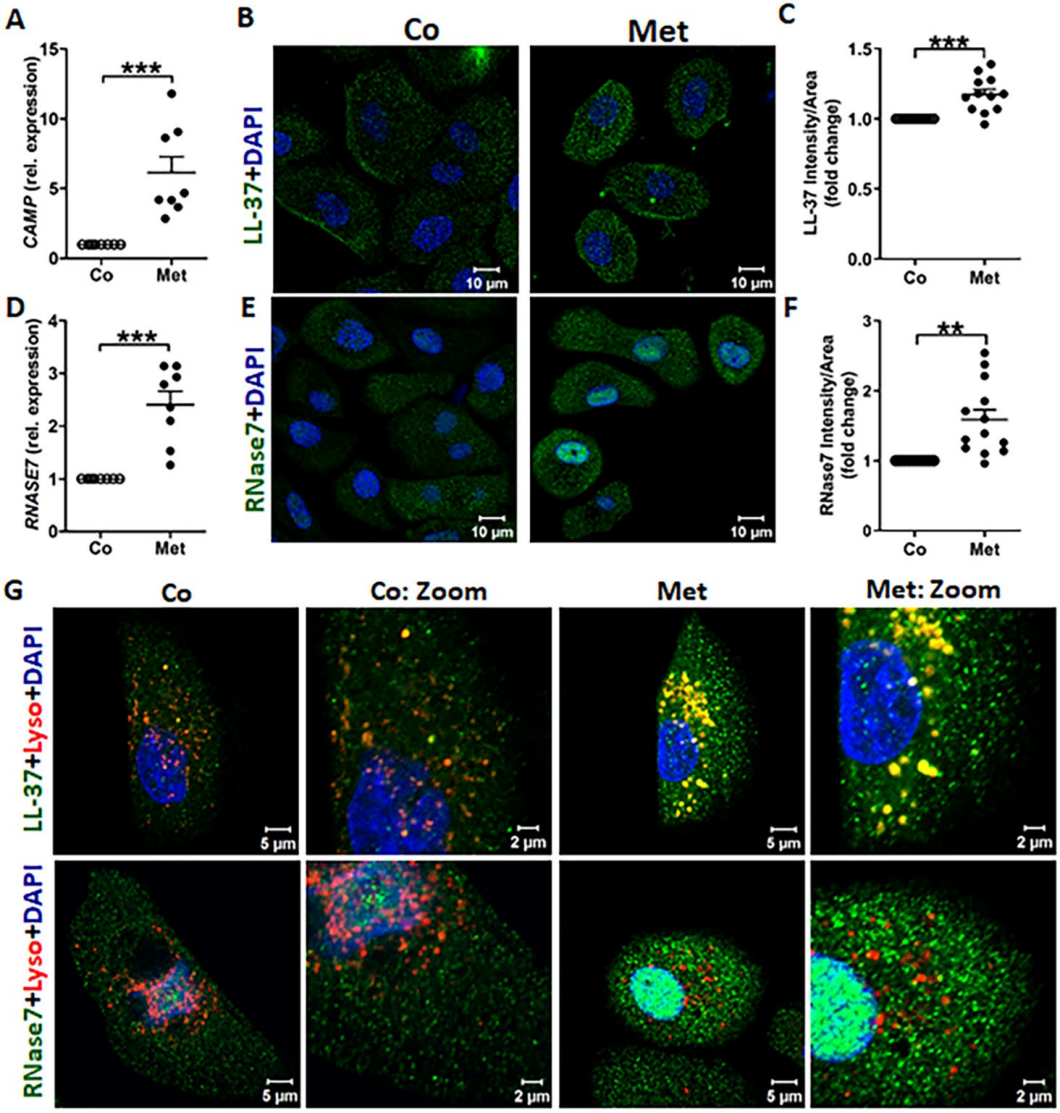
Metformin increases the expression of antimicrobial peptide LL-37 and RNase7 in human uroepithelial cells. (**A**) mRNA expression of *CAMP* measured using RT-PCR (n = 4, performed in duplicates). (**B**) Representative microscopic images depicting LL-37 stained with Alexa fluor 488 (green), nucleus stained by DAPI (blue) and (**C**) relative average intensity of LL-37 (n = 3, each consisting of 4–5 random view fields). (**D**) mRNA expression of *RNASE7* (n = 4, performed in duplicates). (**E**) Representative microscopic images of RNase7 (green) and nucleus (blue). (**F**) Relative average intensity of RNase7 (n = 3, each consisting of 4–5 random view fields). (**G**) Representative microscopic images depicting co-localization of LL-37 (upper panel, green) and RNase7 (lower panel, green) with lysosomes (red) (n = 3). All experiments were performed in human uroepithelial cells, TERT-NHUC and treated with metformin (Met, 4 mM) for either 24 h or 36 h for mRNA and protein respectively, compared to only vehicle treated control. Data are presented as mean with SEM. Significance levels **P < 0.01; ***P < 0.001.

Metformin treatment upregulated the expression of the AMPs *CAMP* and *RNASE7* mRNA and corresponding intracellular proteins LL-37 and RNase7, compared to untreated uroepithelial cells, TERT-NHUC (Fig. 1A–F; Fig. S1A–D) and 5637 (Fig. S1E–J). 4 mM metformin treatment was found to be optimal based on the dose response profiles of *CAMP* and *RNASE7* expression in both TERT-NHUC and 5637 (Fig. S1A,B,E,H). The intracellular activity of AMPs, especially within the lysosomes is vital in the uroepithelial cells, as *E. coli* can neutralize the lysosomal pH, thus escaping the autophagy machinery^16^. Interestingly, metformin treatment resulted in increased localization of LL-37, but not RNase7 in the lysosomes of TERT-NHUC (Fig. 1G) and 5637 cells (Fig. S1K), as shown by confocal microscopy. Furthermore, expression of LL-37 and RNase7 was confirmed by flow cytometry (Fig. S1C,D). The differential expression and localization of these AMPs in uroepithelial cells could be regulated by different signaling pathways triggered by metformin.

### Metformin upregulates CAMP and RNASE7 expression via distinct mechanisms

**Figure 2:**
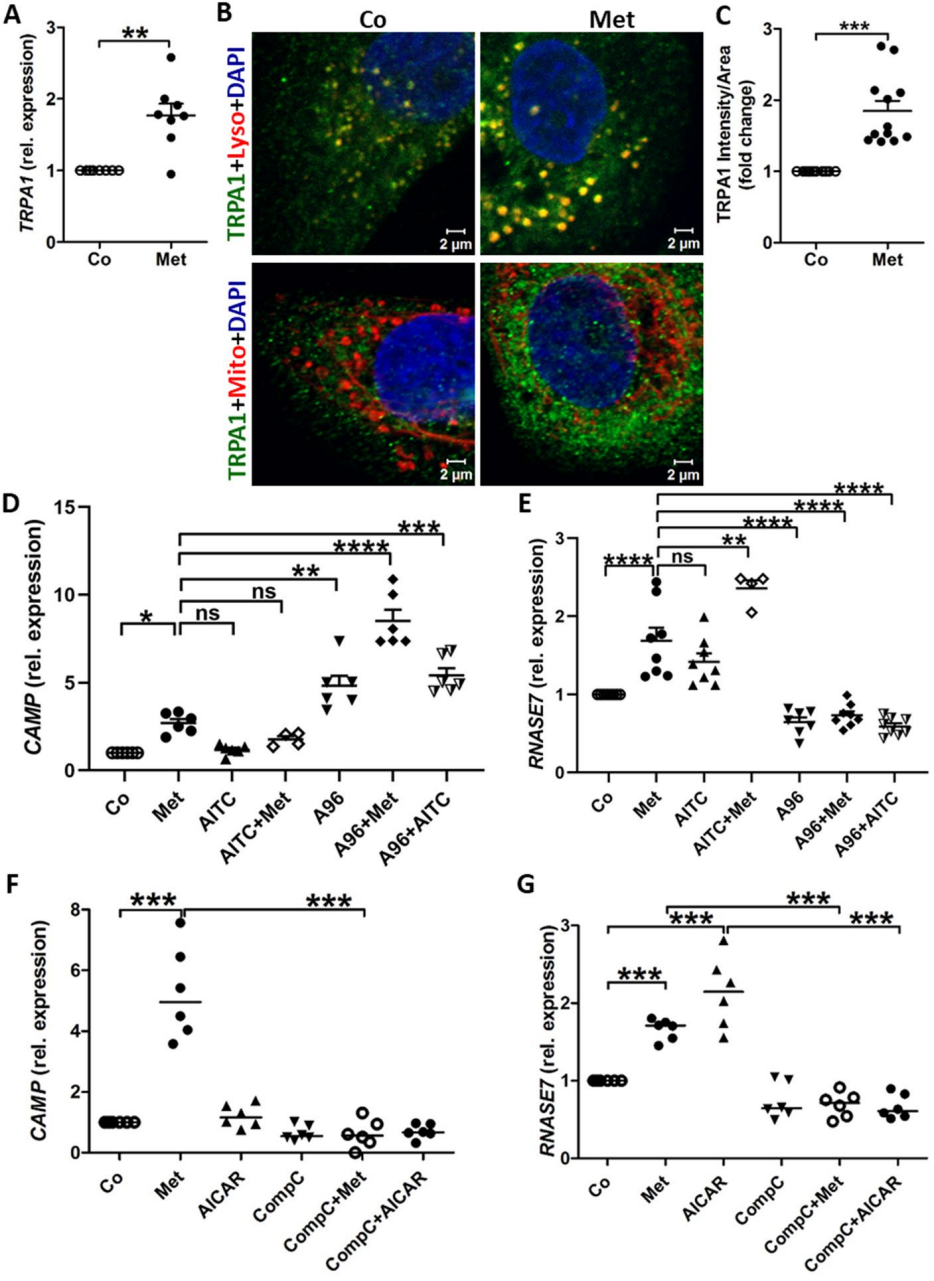
TRPA1 channel and AMPK modulation by metformin upregulates *CAMP* and *RNASE7* expression. (**A**) Expression of *TRPA1* mRNA (n = 4, performed in duplicates) and (**B**) TRPA1 protein upon metformin (Met, 4 mM) treatment vs untreated cells stained for either lysosome or mitochondria (red) (n = 2 independent experiments), with (**C**) quantification of TRPA1 protein expression (n = 3 independent experiments, each consisting of 4 random view fields). (**D**,**E**) Expression of *CAMP* and *RNASE7* upon treatment with metformin (4 mM), AITC (10 µM), AITC together with metformin, A96 (20 µM), A96 pretreatment followed by metformin or A96 pretreatment followed by AITC (n = 3, performed in duplicates). (**F**,**G**) Expression of *CAMP* and *RNASE7* upon treatment with metformin (Met, 4 mM), AMPK activator, AICAR (1 mM) or AMPK inhibitor, compound C (CompC, 20 µM), Compound C pretreatment followed by metformin or Compound C pretreatment followed by AICAR (n = 3, performed in duplicates). All experiments were performed in human uroepithelial cells, TERT-NHUC, treated with respective drugs for 24 h for mRNA and 36 h for protein expression, compared to only vehicle treated control. Data are presented as mean with SEM. Significance levels *P < 0.05, **P < 0.01, ***P < 0.001, ****P < 0.0001.

Recently metformin has been suggested to act on TRPA1 channels^17^. TRPA1 is known to be present in bladder epithelial cells^18^ and is activated upon binding to the *E. coli* lipopolysaccharide^19^. Similar to uroepithelial cells, TRPA1 is also active at the lysosomes of the dorsal root ganglion neurons^20,21^. We therefore investigated if uroepithelial cells also respond to metformin stimulation via TRPA1. Metformin treatment did not increase the intracellular calcium levels in TERT-NHUC, whereas TRPA1 activation by Allyl Isothiocyanate (AITC) increased intracellular calcium (Fig. S2A,B; Videos 1, 2). Pretreatment of TERT-NHUC with either metformin or TRPA1 inhibitor A-967079 (A96) prevented the AITC induced calcium influx (Fig. S2C,D; Videos 3, 4). This indicated that metformin acts as a TRPA1 inhibitor in uroepithelial cells, consistent with a previous report in neurons^17^. Metformin upregulated TRPA1 mRNA and protein expression in TERT-NHUC, with prominent enrichment of TRPA1 specifically in the lysosomes but not in other organelles such as mitochondria (Fig. 2A–C). In addition, the TERT-NHUC showed significant upregulation of *CAMP* upon treatment with metformin or TRPA1 inhibitor A96, and additive effect on treating A96 pretreated cells with metformin (Fig. 2D). TRPA1 activation by AITC itself had no impact on *CAMP* expression (Fig. 2D). Furthermore, AITC was not capable of blocking *CAMP* expression induced by pretreatment with TRPA1 inhibitors metformin or A96, as the ion channels were not activated in the presence of their inhibitors. In contrast, AITC treated TERT-NHUC showed a trend of increased *RNASE7* expression and an additive increase of *RNASE7* was observed upon treatment with both metformin and AITC (Fig. 2E). A96 itself was able to decrease *RNASE7* expression, while A96 pretreatment blocked the metformin and AITC induced increase in *RNASE7* expression (Fig. 2E). Thus, *CAMP* expression was regulated by TRPA1 channel inhibition, while *RNASE7* expression was likely regulated by a mechanism that is triggered by metformin and TRPA1 activation, probably by AMPK activation. These findings are in line with our calcium imaging data (Fig. S2) and previous reports showing that metformin acts as an inhibitor of TRPA1^17^.

Metformin is a well-established activator of the AMPK pathway^22^, however it is not known to stimulate *CAMP* or *RNASE7* expression. In the uroepithelial cells, TERT-NHUC treated with metformin and AMPK modulators, *CAMP* expression remained unaffected upon AMPK activation by AICAR (5-aminoimidazole-4-carboxamide ribonucleotide) or inhibition by compound C (Fig. 2F). On the other hand, *RNASE7* was significantly upregulated upon AMPK activation by both metformin and AICAR (Fig. 2G), confirming the direct involvement of the AMPK pathway. Interestingly, pretreatment with the AMPK inhibitor compound C prevented metformin induced upregulation of both *CAMP* and *RNASE7*. These results suggest that metformin induced *CAMP* expression is regulated by inhibition of TRPA1 channels while *RNASE7* expression is mediated by the activation of the AMPK pathway. In addition to these antimicrobial peptides, other innate immune mechanisms might also be triggered by metformin.

### Free radicals are induced by metformin

**Figure 3:**
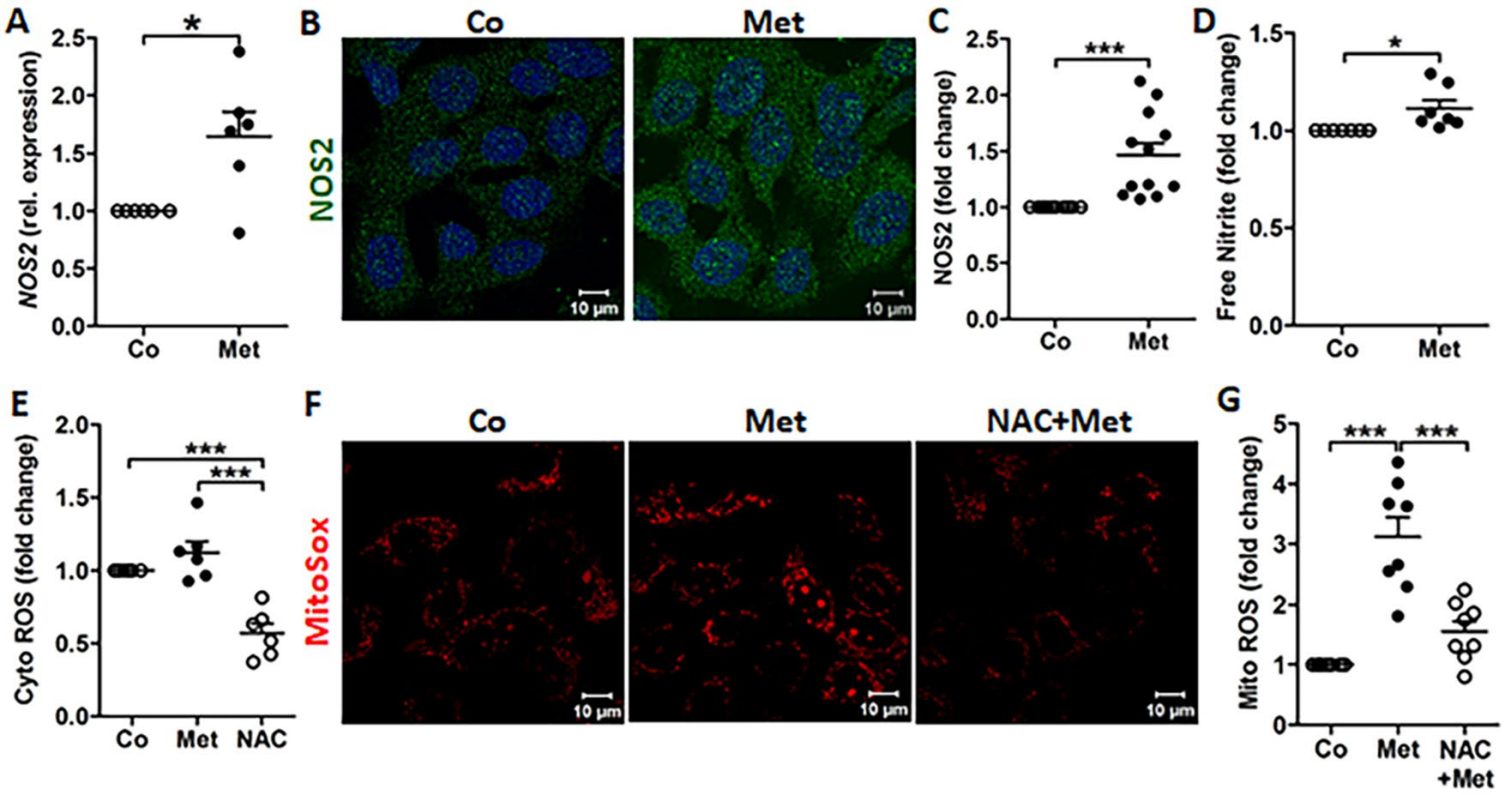
ROS and NO production are stimulated by metformin. (**A**) Expression of *NOS2* mRNA (n = 3, performed in duplicates), (**B**) representative images depicting expression of NOS2 enzyme stained with Alexa fluor 488 (green), nucleus stained by DAPI (blue) and (**C**) relative average intensity of NOS2 (n = 3, each consisting of 4 random view-fields). (**D**) Free nitrite was measured in the conditioned media by Griess reagent (n = 4, performed in duplicates). (**E**) Measurement of cytosolic ROS using H2DCFDA, in metformin (Met, 4 mM) or ROS inhibitor *N*-acetyl-l-cysteine (NAC, 3 mM) treated, untreated cells (n = 3, performed in duplicates). (**F**) Representative mitochondrial ROS images depicting MitoSox Red staining in untreated, metformin or NAC + metformin treated cells (n = 2). (**G**) Average relative intensity of MitoSox Red (n = 2, each consisting of 4 random view-fields). Human uroepithelial cells, 5637 treated with indicated drugs for 24 h. Data are presented as mean with SEM. Significance levels *P < 0.05, ***P < 0.001.

While few studies have reported the effect of metformin on lysosomes^23^, metformin is believed to act primarily on mitochondrial respiration^24^. Mitochondria are a source of free radical species including reactive oxygen species (ROS) and reactive nitrogen species (RNS)^25^. Besides AMPs, the uroepithelial cells also produce ROS and RNS which help to control invading pathogens^26^. In uroepithelial cells, 5637, metformin treatment increased nitric oxide synthase (*NOS2*) mRNA and protein expression (Fig. 3A–C), along with higher release of free nitric oxide (Fig. 3D), without altering the expression of the human antioxidant genes catalase (*CAT*) and superoxide dismutase (*SOD2)* (Fig S3A, B). Notably, metformin treatment showed specific increase in mitochondrial, but not cytosolic ROS (Fig. 3E–G), which was decreased by pretreatment with the ROS inhibitor *N*-acetyl-l-cysteine (NAC). This is in agreement with previous reports attributing the antimicrobial activity of metformin to increased mitochondrial ROS production in macrophages^13,14^.

### Metformin promotes bacterial clearance

**Figure 4:**
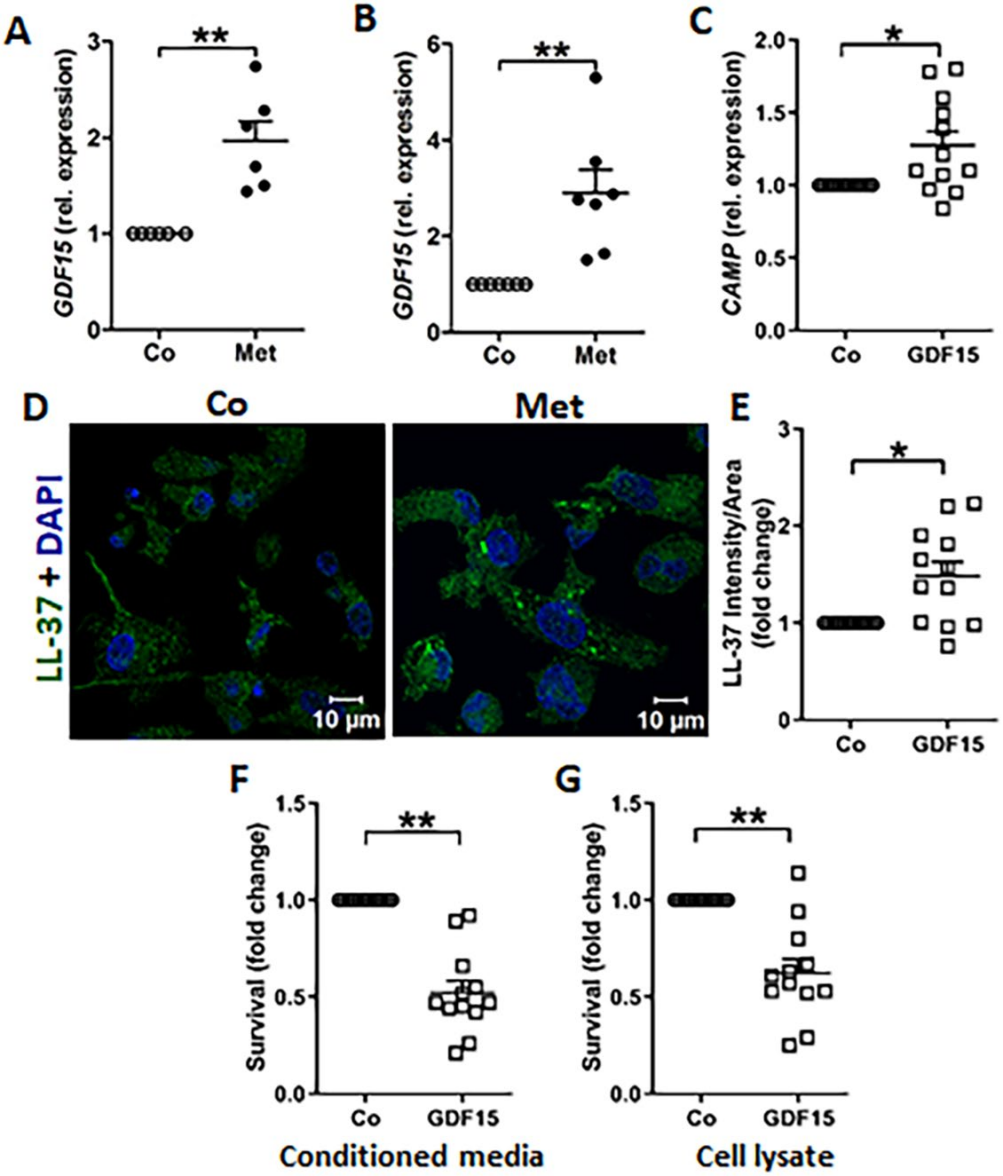
Metformin induces GDF15 expression, with increased antibacterial activity in macrophages. Expression of *GDF15* mRNA in (**A**) 5637, (**B**) TERT-NHUC uroepithelial cells (n = 3, performed in duplicates) after treatment with metformin (Met, 4 mM). (**C**–**E**) Expression levels of LL-37 mRNA (n = 4, performed in triplicates) and protein in THP1 macrophages treated with GDF15 peptide (50 ng/ml) for 24 or 36 h respectively, followed by quantification of LL-37 expression (n = 3, each consisting of 4 random view fields). *E. coli* survival after (**F**) 6 h incubation in conditioned media or (**G**) 30 min incubation in cell lysate from metformin treated THP1 cells (n = 4). Data are presented as mean with SEM. Significance levels *P < 0.05, **P < 0.01.

Interestingly, metformin was recently shown to upregulate the peptide hormone, growth/differentiation factor 15 (GDF15)^27,28^ which was demonstrated to support host survival during bacterial infection and sepsis^29^. Notably, metformin treated uroepithelial cells upregulated *GDF15* mRNA expression in both 5637 (Fig. 4A) and TERT-NHUC (Fig. 4B). GDF15 expression was upregulated by metformin, TRPA1 inhibitor A96 (Fig. S4A) and AMPK inhibitor compound C (Fig. S4B). Neither AITC nor AICAR showed upregulation in *GDF15* expression. However, we did not observe any direct antibacterial effect of GDF15 peptide on uropathogenic *E. coli* (data not shown). Treatment of uroepithelial cells with GDF15 peptide did not increase *CAMP* or *RNASE7* expression in 5637 (Fig. S4C,D) or TERT-NHUC cells (Fig. S4E,F). The induction of these AMPs in uroepithelial cells therefore seems to be GDF15 independent. This observation raised the possibility that GDF15 produced by uroepithelial cells stimulates other immune cells like macrophages. Interestingly, GDF15 peptide treatment upregulated both mRNA and protein expression of LL-37 in human THP1 monocyte derived macrophages (Fig. 4C–E). In line with this finding, increased bacterial killing was observed in the conditioned media and cell lysate of GDF15 peptide stimulated macrophages (Fig. 4F,G).

**Figure 5:**
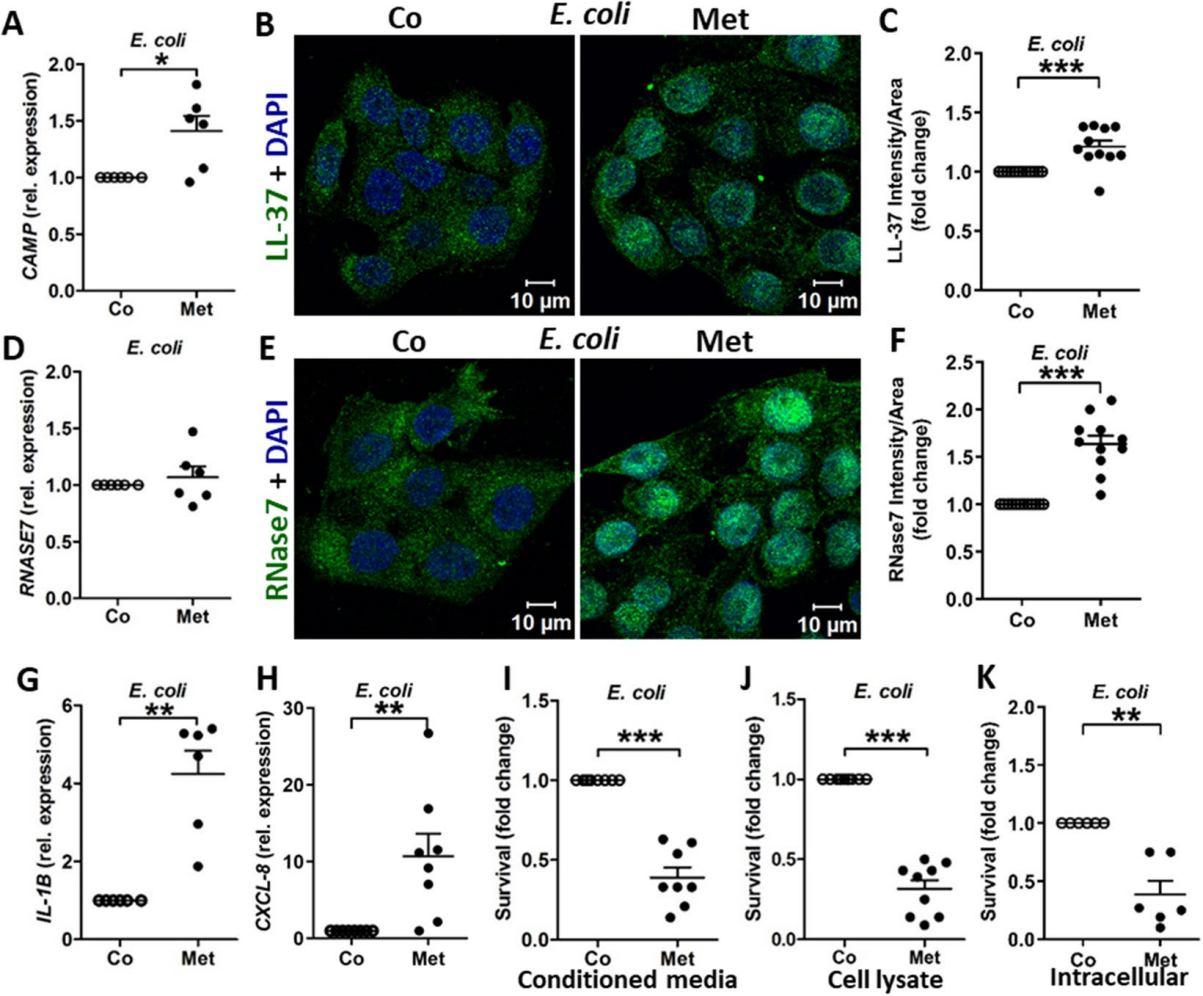
Metformin treated uroepithelial cells show higher antibacterial activity. (**A**) Expression of *CAMP* in 5637 uroepithelial cells measured after *E. coli* infection at MOI 20 for 15 min (n = 3, performed in duplicates). (**B**) Representative microscopic images of LL-37 expression after 2 h *E. coli* infection with MOI 10 and (**C**) relative average intensity analysis (n = 3, each consisting of 3–4 random view fields). (**D**–**F**) Corresponding expression pattern of RNase7 at mRNA and protein level. (**G**,**H**) Expression of *IL1B*, *CXCL8* in infected 5637 uroepithelial cells (n = 3–4, performed in duplicates). (**I**) *E. coli* survival after 6 h incubation in uroepithelial cells conditioned media and (**J**) 30 min in the cell extract (n = 3). (**K**) Intracellular survival of *E. coli* after 2 h post gentamicin treatment and compared to initial inoculum (n = 3). All survival experiments were performed in duplicates or triplicates. Human uroepithelial cells, 5637 were used throughout. Metformin (Met, 4 mM) treatment for 24 or 36 h for mRNA and protein analysis or survival assays respectively, compared to only vehicle treated control. Data are presented as mean with SEM. Significance levels *P < 0.05, **P < 0.01, ***P < 0.001.

Since uropathogenic *E. coli* are known to invade uroepithelial cells and survive intracellularly^4,30^, we explored whether metformin pretreated cells were better prepared to fend off invading bacteria. Upon infection with uropathogenic *E. coli*, metformin treated 5637 and TERT-NHUC cells showed upregulation in LL-37 at the mRNA and protein levels (Fig. 5A–C; Fig. S5A–C). Similarly, RNase7 protein expression also increased in 5637 cells upon infection, though only mRNA upregulation was observed in TERT-NHUC (Fig. 5D–F; Fig. S5D–F).

AMPs are also known to stimulate proinflammatory cytokines which help in the recruitment of immune cells such as macrophages, neutrophils and NK cells that promote resolution of bladder infection^31,32^. Metformin treated 5637 cells increased mRNA expression of the proinflammatory cytokines *IL1B* and *CXCL-8* upon infection (Fig. 5G,H).

Further, to explore the effect of metformin in inhibiting bacterial growth in uroepithelial cells, survival assays were performed in conditioned media, cell lysates and intact cells. Significantly increased bacterial killing was observed in the conditioned media from metformin treated 5637 (Fig. 5I) and TERT-NHUC cells (Fig. S5G). Interestingly, the cell lysates from metformin treated uroepithelial cells retained higher antimicrobial activity (Fig. 5J, Fig. S5H). Finally, metformin also increased intracellular *E. coli* killing in uroepithelial cells, 5637 (Fig. 5K) and TERT-NHUC (Fig. S5I) compared to untreated cells.

## Discussion

We demonstrate that metformin exerts its effect through different host protective mechanisms, prominent being upregulation of the AMPs, LL-37 and RNase7. The AMPs are particularly interesting antibacterial agents as they can act intracellularly as well as extracellularly. Moreover, their intracellular localization, especially within the lysosomes are vital. LL-37 exerts its activity against intracellular pathogens upon enrichment in lysosomes of epithelial cells^33^ as well as in macrophages^34^. In vitro metformin treatment induced lysosomal enrichment of LL-37 could therefore better prepare uroepithelial cells against bacterial invasion. Interestingly, RNase7 was not localized in the lysosomes upon metformin treatment. This is in line with a previous report demonstrating that RNase7 does not accumulate in lysosomes, though its related antimicrobial peptide RNase6 can localize to lysosomes^35^. It is worthy of note that metformin also increased TRPA1 expression in the lysosomes of uroepithelial cells. Our calcium imaging, together with previous reports demonstrates that TRPA1 is active in the lysosomes^20,21^.

Lysosomes also act as a hub of AMPK activity^36^, which is crucial for lysosomal biogenesis and function^37^. Hence, we explored the role of metformin mediated AMPK activation in regulating *CAMP* and *RNASE7* expression. While *RNASE7* expression was AMPK-dependent, *CAMP* expression was largely AMPK-independent. Despite the fact that compound C can inhibit several other kinases^38^, we observed that compound C itself had no effect on *RNASE7* expression. Compound C significantly blocked metformin and AICAR induced *RNASE7* expression, suggesting that metformin induced *RNASE7* expression is dependent on AMPK activation. On the other hand, *CAMP* expression is dependent on TRPA1 inhibition by metformin or TRPA1 specific inhibitor A96. These evidences point towards activation of at least two distinct pathways governing *CAMP* and *RNASE7* induction by metformin.

Metformin can increase the antimicrobial effect of macrophages further by direct stimulation and via immunomodulatory agents produced by uroepithelial cells. GDF15 is one such host protective molecule, which is upregulated by metformin and is protective against bacteria induced sepsis^29^. Metformin and A96 upregulated GDF15 mRNA expression, indicating that TRPA1 inhibition can increase GDF15 expression. Notably, this mechanism is similar to the induction of *CAMP* by TRPA1 inhibition. However, treatment of uroepithelial cells with GDF15 peptide did not impact *CAMP* or *RNASE7* expression. Hence, the expression of these antimicrobial peptides is independent of GDF15 in uroepithelial cells. Metformin induced GDF15 might influence multiple host protective mechanisms. It is likely that metformin induced GDF15 in uroepithelial cells improves the antibacterial efficacy of other innate immune cells such as macrophages by stimulating antimicrobial peptide production.

Most importantly, both the uroepithelial cells tested, TERT-NHUC and 5637, showed significantly improved killing of intracellular and extracellular bacteria upon metformin treatment, indicating that metformin prepares these uroepithelial cells to kill pathogenic bacteria more efficiently. The increase in *CAMP* and *RNASE7* mRNA levels during infection of metformin treated uroepithelial cells could be attributed to rapid transcriptional activation^2^. The significant upregulation of LL-37 peptide, which was not observed for RNase7 in metformin treated and infected TERT-NHUC uroepithelial cells, suggest differential translational regulation of these AMPs. Most of the cytosolic pool of AMPs are released to the extracellular medium when the bacteria contact the plasma membrane of uroepithelial cells. Hence, higher transcriptional induction of AMPs in metformin pretreated cells upon infection, could be advantageous for host defense.

Taken together, our data demonstrate that metformin induces multiple antimicrobial mechanisms in uroepithelial cells. The enhanced bacterial clearance by metformin treated human uroepithelial cells is most likely influenced by the combined action of increased innate immune response including AMPs, ROS, NOS and cytokines. Therefore, in the future reorienting drugs that also strengthen host mediated antibacterial effects, like metformin, can be of interest to complement traditional treatment strategies.

## Materials and methods

### Cell lines and culture conditions

Telomerase-immortalized human uroepithelial cells, TERT-NHUC (provided by M. A. Knowles, Leeds, UK) and human bladder epithelial cell line, 5637 (HTB-9, American Type Culture Collection) were cultured as previously described^4^. In brief, TERT-NHUC cells were grown in Epilife medium (MEPI500CA, Life Technologies) supplemented with 1% of human keratinocyte growth supplement (S0015, Life technologies) and 5637 cells, human monocytes THP1 (TIB-202, ATCC) were grown in RPMI 1640 medium (Life Technologies) supplemented with 10% fetal bovine serum (Life Technologies), and cultured at 37 °C and 5% CO_2_. THP1 monocytes were differentiated to macrophages by treatment with 150 ng/ml of phorbol myristate acetate (PMA) for 24 h followed by replacement with PMA-free RPMI 1640 medium for another 24 h.

### Bacteria

Uropathogenic *E. coli* strain CFT073 was cultured on blood agar plates overnight, then grown to logarithmic phase in Luria Bertani broth with shaking at 37 °C. The bacterial concentration was adjusted to 10^8^ CFU/ml in phosphate-buffered saline (PBS) spectrophotometrically and confirmed by viability count.

### Cell treatment

In vitro cell experiments were carried out in Primaria plates (BD Falcon) for uroepithelial cells, TERT-NHUC, and polystyrene (Costar) for 5637 and THP1 differentiated cells. Nearly confluent cells, 70–80%, were treated with freshly prepared metformin (4 mM, Sigma-Aldrich) for 24 h, deionized water served as vehicle control. 4 mM metformin concentration was found to be optimal for both the cell lines based on the dose response expression profiles of *CAMP* and *RNASE7* expression (Fig. S1).

5' adenosine monophosphate-activated protein kinase (AMPK) activator 5-Aminoimidazole-4-carboxamide ribonucleotide (AICAR, 1 mM, Sigma-Aldrich) or AMPK inhibitor, Compound C (20 µM, Sigma-Aldrich) were used and cells were treated for 24 h. In blocking experiments, Compound C was added 1 h prior to adding AICAR or metformin. Chinese hamster ovary cell line expressed growth/differentiation factor 15 peptide (GDF15, 50 ng/ml, R&D systems) was treated to the cells for 24 h. For TRPA1 RT-PCR or imaging experiments, cells were stimulated with the activator, allyl isothiocyanate (AITC, 10 µM, Sigma-Aldrich) and inhibitor A-967079 (A96, 20 µM, Sigma-Aldrich), for 24 h for mRNA analysis. Equal amount of DMSO served as vehicle in control cells.

### Total RNA extraction and quantitative real-time PCR

Total RNA extraction was performed as previously described^7^ and up to 500 ng of RNA was transcribed to complementary DNA with the Superscript cDNA Synthesis Kit (Life Technologies) according to the manufacturers’ instructions. Gene expression was analyzed by SYBR Green-based assay (Life Technologies) in a Rotor-Gene PCR cycler (Corbett Life Science) using gene specific primers: (*CAMP* forward 5’-ACC CAG CAG GGC AAA TCT-3’, *CAMP* reverse 5’-GAA GGA CGG GCT GGT GAA-3’, *RNASE7* forward 5’-GGA GTC ACA GCA CGA AGA CCA-3’, *RNASE7* reverse 5’-CAT GGC TGA GTT GCA TGC TTG A-3’, *IL1B* forward 5’- CAC GAT GCA CCT GTA CGA TCA-3’, *IL1B* reverse 5’-GTT GCT CCA TAT CCT GTC CCT-3’, *CXCL8* forward 5’-AAG AGA GCT CTG TCT GGA CC-3’, *CXCL8* reverse 5’-GAT ATT CTC TTG GCC CTT GG-3’, *TRPA1* forward 5’-GAA ACC AAA GTG GCA GCT TC -3’, *TRPA1* reverse 5’-GAC ATT GCT GAG GTC CAG AA -3’, *GDF15* forward 5’-GTGTTGCTGGTGCTCTCGTG -3’, *CAT* forward 5’- GTG CGG AGA TTC AAC ACT GCC A-3’, *CAT* reverse 5’- CGG CAA TGT TCT CAC ACA GAC G-3’, *SOD2* forward 5’- CTG ATT TGG ACA AGC AGC AA-3’, *SOD2* reverse 5’- CTG GAC AAA CCT CAG CCC TA-3’, *GDF15* forward 5’-GTGTTGCTGGTGCTCTCGTG -3’, *GDF15* reverse 5’-CGGTGTTCGAATCTTCCCAG -3’, *ACTB* forward 5’- AAG AGA GGC ATC CTC ACC CT -3’, *ACTB* reverse 5’-TAC ATC GCT GGG GTG TTG-3’). *ACTB* served as housekeeping control. Relative expressions of target genes were presented as 2^−∆CT^ and fold change as 2^−∆∆CT^ compared to non-treated control.

### Immunofluorescence analysis

Uroepithelial cells were grown on glass cover-slips (VWR) and treated as described above. Cells were processed for immuno-staining as previously described^39^ using primary antibody against LL-37 (Santa Cruz Biotechnology, produced in mouse), RNase7 (Novus, produced in rabbit), TRPA1 (Alomone Labs, produced in rabbit) at 1:200 dilution and secondary anti-mouse or anti-rabbit Alexa Fluor dye–conjugated antibodies (Invitrogen) at 1:400 dilution. Where indicated, cells were stained with LysoTracker Red DND-99 (1 µM, 30 min, Invitrogen) to visualize the lysosomes. Imaging was performed using 63X oil immersion objective of Zeiss LSM700 confocal microscope (Carl Zeiss, Germany). Fluorescence intensity and area of each cell was quantified by manually defining the boundaries around each cell with the ImageJ software (U. S. National Institutes of Health). For each cell, the intensity/area ratio was calculated. Since each view-field contained 4–5 cells, the average of intensity/area values of all cells in the view-field was calculated. For each view-field, the intensity of the target was normalized to the average intensity of the control. H ence, each dot in the presented graph represents average intensity/area values of one view-field. 4 view-fields were quantified from 3 independent experiments. Therefore, the graphs represent data from approximately 5 × 4 × 3 = 60 cells but have 12 values (representing each view-field).

### Flow cytometry

Uroepithelial cells, TERT-NHUC were either untreated or treated with or without metformin (4 mM, 8 mM) for 36 h, and harvested by standard trypsinization method. Cells were stained for viability with LIVE/DEAD™ Fixable Aqua Dead Cell Stain Kit (Invitrogen), diluted 1:50 in PBS for 30 min at 4 °C followed by washing in PBS with 1% FBS. The cells were then fixed with 4% PFA at RT for 15 min and permeabilized with Perm buffer (BD Biosciences). Thereafter the cells were stained with mouse LL-37 and rabbit RNase7 (1:200, diluted in Perm buffer) for 30 min at 4 °C. Afterwards, cells were washed and stained in presence of Perm buffer with respective Alexa fluor goat anti-mouse 488 and donkey anti-rabbit 647 (1:400, diluted in Perm buffer) for 20 min at RT. The cells were then washed and resuspended in PBS and at least 6,000–20,000 cells were acquired using LSRFortessa™ flow cytometer and analyzed using BD FACSDiva™ software (BD Biosciences Immunocytometry Systems). Control staining with only secondary antibodies was performed to exclude potential unspecific binding. Data are presented as fold change of mean fluorescence intensity of the antimicrobial peptides.

### Calcium imaging

TERT-NHUC cells were grown on glass bottom dishes (ibidi), treated with 2 µM of the membrane permeant Ca^2+^ indicator dye Fluo-4 AM (Invitrogen) for 30 min or 1 µM of LysoTracker red (Invitrogen) for 30 min. After indicated time, cells were washed with HBSS, freshly supplemented with medium and time series imaging was performed in green or red channel using 63 × oil immersion objective of Zeiss LSM700 confocal microscope for 300 frames at 1.56 s/frame interval. Drugs were administered to the imaging dish at the time points indicated in the graphs.

### Reactive Oxygen Species measurements

Uroepithelial cell, 5637 were treated with metformin (4 mM) with or without 1 h pretreatment with the ROS chelator N-acetyl cysteine (NAC, 3 mM Sigma Aldrich) for 24 h. Cells were stained with MitoSox Red (5 µM, Invitrogen) for 30 min and images were captured in LSM700 confocal microscope.

For cytosolic ROS measurements, uroepithelial cells, 5637 were treated with metformin (4 mM) or NAC for 24 h. Cytosolic ROS was quantified by staining the cells with 2',7'-dichlorodihydrofluorescein diacetate (H2DCFDA, 10 µM, Sigma Aldrich) and fluorescence signal quantified using Fluorimeter (Omega) at excitation of 485 nm and emission 530 nm. Data were represented as relative fold change with respect to untreated control.

### Nitric oxide measurement

Nitric oxide in the cell culture supernatants from untreated 5637 cells and cells treated with metformin (4 mM) for 24 h were incubated with Griess reagent for 10 min at room temperature, followed by absorbance recording at 540 nm.

### Conditioned media and antimicrobial killing assay

Cell free supernatants were collected from metformin (4 mM) treated uroepithelial cells after 36 h incubation. Cell extracts were prepared by lysing the cells in 1% Triton-X-100, centrifuged at 8000 g for 5 min to remove cell debris. 50 µl from 10^4^ CFU/ml of *E. coli* were added to 150 µl of conditioned media or cell extract and incubated at 37 °C CO_2_ at 150 rpm. After 30 min incubation, 100 µl of cell extract was plated, while 50 µl of conditioned media was cultured after 6 h of incubation for enumerating the CFU.

### Intracellular survival assay

For intracellular survival assays, uroepithelial cells 5637 and TERT-NHUC were infected with *E. coli* at 1:10 MOI for 2 h, without changing media, followed by washing with 1X PBS and replaced with fresh media for another 2 h. Infected cells were vigorously washed with 1X PBS to get rid of extracellular bacteria. Cells were lysed with 0.1% Triton X-100 in PBS, serially diluted in 1X PBS and viable count was performed. Percentage of bacterial survival was calculated with respect to pre-inoculation density.

### Statistical analysis

Data are presented as individual values with mean ± SEM. Statistical outliers, defined by Grubb’s test were excluded from the analysis. Data involving two variables were compared by Mann Whitney test, while those involving multiple variables were compared by ANOVA. Differences with *P* < 0.05 were regarded as statistically significant. All statistical tests were performed with GraphPad Prism versions 5.02 and 8.3.0.

## Supplementary Information

Supplementary Video 1.

Supplementary Video 2.

Supplementary Video 3.

Supplementary Video 4.

Supplementary Information 1.

## Data Availability

The datasets generated during and/or analyzed during the current study are available from the corresponding author on reasonable request.
