# Supplementary material for: Metformin strengthens uroepithelial immunity against E. coli infection
Source: Sci Rep. 2021 Sep 28;11:19263. doi: 10.1038/s41598-021-98223-1 (PMC8479095; doi:10.1038/s41598-021-98223-1)
Supplement: Supplementary file 5 — Supplementary Information 1. [file 41598_2021_98223_MOESM5_ESM.docx]

*Supplementary file*

**Metformin strengthens uroepithelial immunity against *E. coli* infection**

Rakesh Kumar Majhi^1,2^, Soumitra Mohanty^1,2,†^, Witchuda Kamolvit^1,2,†^, John Kerr White^1,2^, Andrea Scheffschick^3^, Hanna Brauner^3,4^, Annelie Brauner^1,2,*^

^1^ Department of Microbiology, Tumor and Cell Biology, Karolinska Institutet, Stockholm, Sweden.

^2^ Division of Clinical Microbiology, Karolinska University Hospital, Stockholm, Sweden.

^3^ Department of Medicine, Karolinska Institutet, Stockholm, Sweden.

^4^ Dermatology and Venereology Clinic, Karolinska University Hospital, Stockholm, Sweden.

†These authors have contributed equally to this work

* Corresponding author:

Annelie Brauner, Department of Microbiology, Tumor and Cell Biology, Division of Clinical Microbiology, Karolinska Institutet and Karolinska University Hospital, 17176 Stockholm, Sweden.

Phone +46 8 51770000, Fax: +46 8 308099

E-mail: [Annelie.Brauner@ki.se](mailto:Annelie.Brauner@ki.se)

ORCID: R.K.M., 0000-0001-6999-1349; S.M., 0000-0003-0919-5062; J.K.W., 0000-0003-3485-5958; A.S., 0000-0002-2848-2617; H.B., 0000-0003-2810-4189; A.B., 0000-0001-5533-6837.


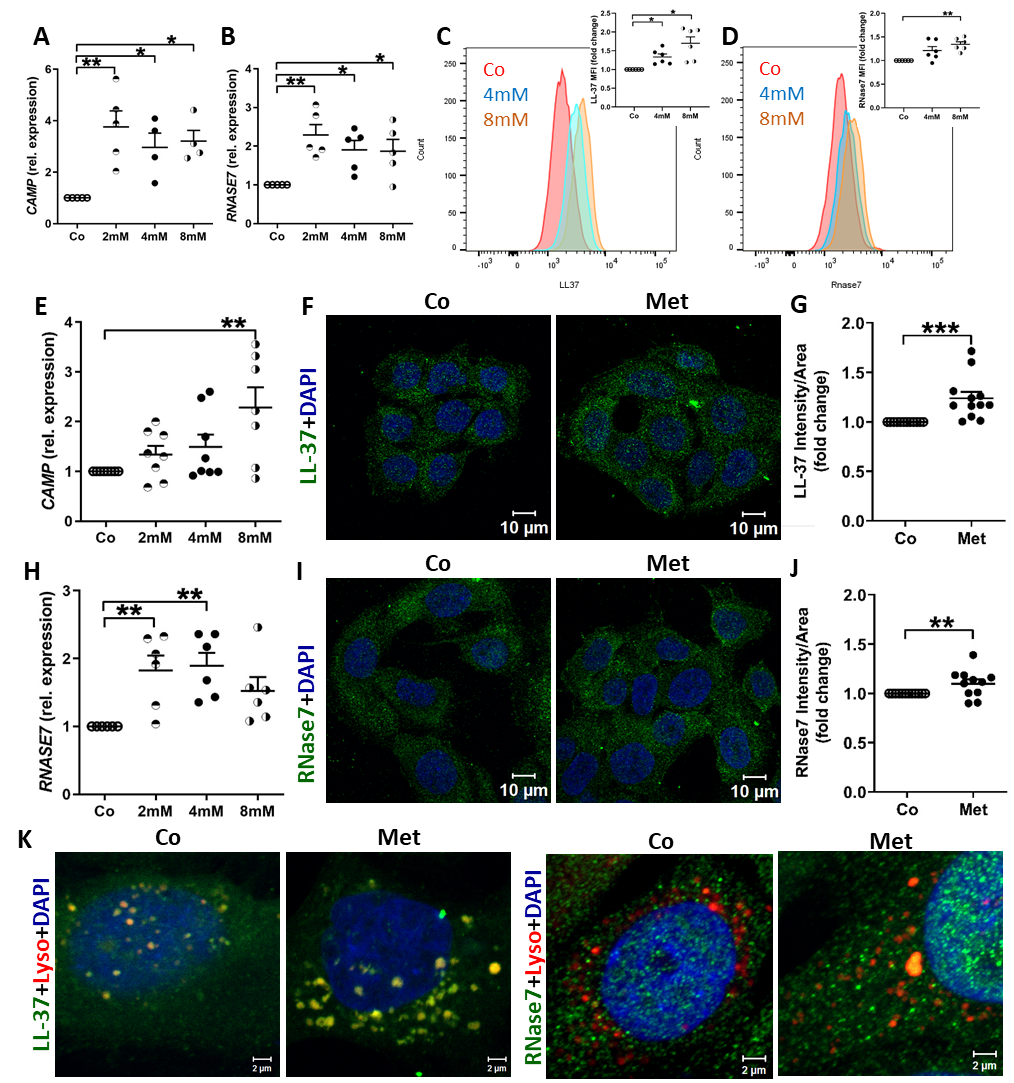


**Figure S1. Metformin increases expression of LL-37 and RNase7 in human uroepithelial cells, 5637.** (A,B) Dose response of metformin treatment on TERT-NHUC at indicated concentrations for 24 h. mRNA expression of *CAMP, RNASE7* was measured using RT-PCR (n=3, performed in singlet/duplicates). (C,D) Dose response of metformin treatment on TERT-NHUC. Expression of antimicrobial peptides LL-37 and RNase7 analyzed by flow cytometry (n=6, performed in singlets). (E) mRNA expression of *CAMP* in 5637 uroepitheilal cells measured using qRT-PCR (n=3, performed in duplicates). (F) Representative microscopic images depicting LL-37 stained with Alexa flour 488 (green), nucleus stained by DAPI (blue) and (G) relative average intensity of LL-37 (n=3, each consisting of 4 random view-fields). (H) mRNA expression of *RNASE7* (n=3, performed in duplicates). (I) Representative microscopic images of RNase7 (green) and nucleus (blue). (J) Relative average intensity of RNase7 (n=3, each consisting of 4 random view-fields). (K) Representative microscopic images depicting co-localization of LL-37 (left panel, green) and RNase7 (right panel, green) with lysosomes (red) (n=3). All experiments were performed in human uroepithelial cells, TERT-NHUC or 5637 and treated with metformin (Met, 4mM) for either 24 h or 36 h for mRNA and protein respectively, compared to only vehicle treated control. Data are presented as mean with SEM. Significance levels *P<0.05; **P<0.01; ***P<0.001.


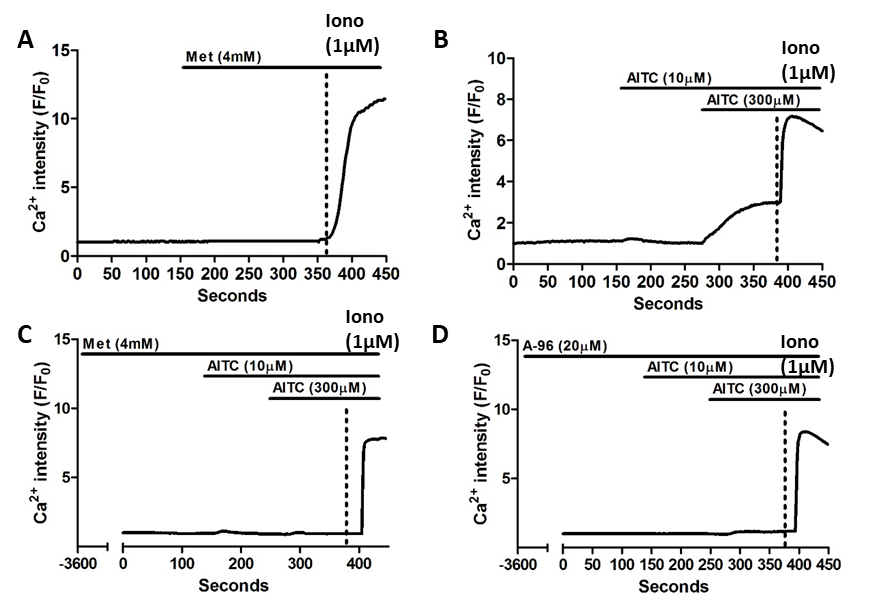


**Figure S2. Metformin inhibits TRPA1 channel.** (A-D) Time-lapse imaging of intracellular Ca^2+^ detected in Fluo-4 AM loaded cells. Each trace depicts ratio of fluorescence intensity at indicated time point normalized to initial intensity. Bars above represent time points at which metformin (Met), TRPA1 activator AITC or TRPA1 inhibitor A-967079 (A96) were added. Average values from 15 cells were presented (n=2; 28-30 cells). All experiments were performed in human uroepithelial cells, TERT-NHUC.


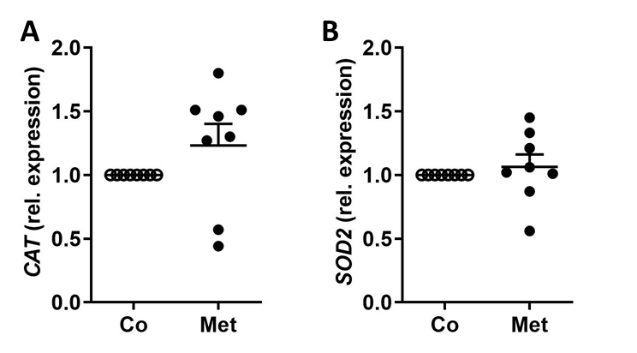


**Figure S3. Metformin does not affect the expression of human antioxidant genes in human uroepithelial cells.** Expression of catalase (*CAT*) and superoxide dismutase (*SOD2)* in 5637 cells treated with metformin (Met, 4mM) for 24 h (n=4, performed in duplicates) relative to the vehicle control (Co). Data are presented as mean with SEM.


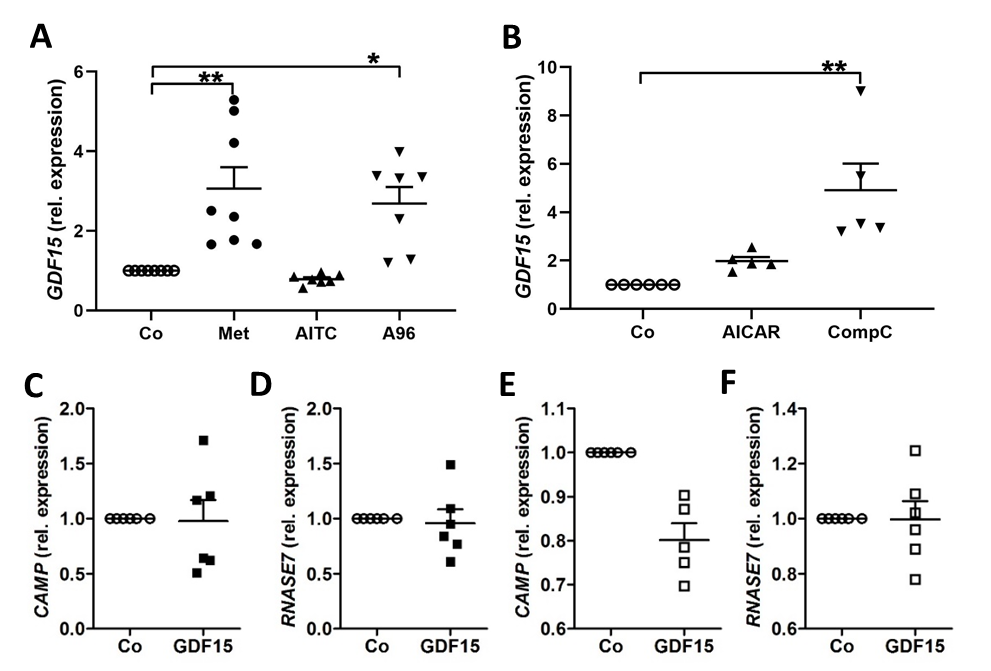


**Figure S4. GF15 peptide expression and its role on AMPs in human uroepithelial cells.** (A). *GDF15* expression in TERT-NHUC uroepithelial cells treated with TRPA1 modulators metformin (Met, 4mM), AITC (10µM), A96 (20µM) (n=4, performed in duplicates). (B) *GDF15* expression in TERT-NHUC uroepithelial cells treated with AMPK modulators AICAR (1mM) or Compound C (CompC, 20µM) (n=3, performed in duplicates). Expression of *CAMP* and *RNASE7* mRNA (C,D) in 5637 and (E,F) in TERT-NHUC cells after treatment with 50ng/ml of GDF15 peptide for 24 h (n=3, performed in duplicates). Data are presented as mean with SEM. Significance levels *P<0.05; **P<0.01.

**
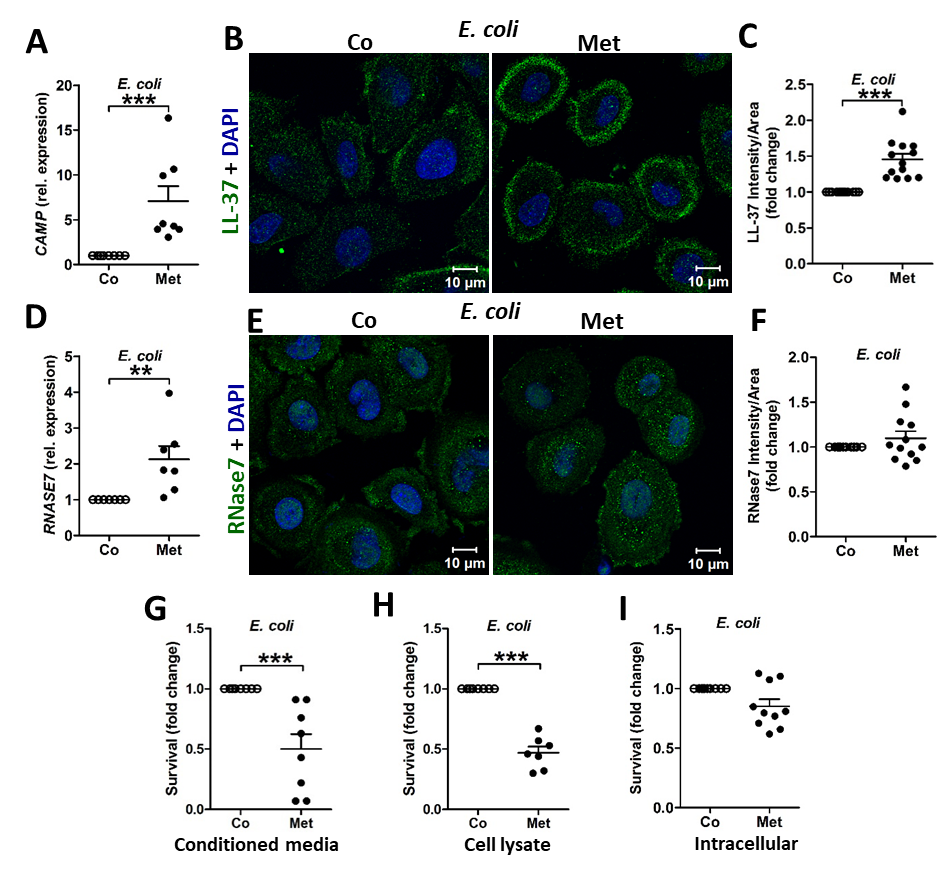
**

**Figure S5. Increased antibacterial activity induced by metformin in human uroepithelial cells.** (A) Expression of CAMP in TERT-NHUC measured after infection with *E. coli* at MOI 20 for 15 min (n=4, performed in duplicates). (B,C) Representative microscopic images of LL-37 expression in TERT-NHUC after 2 h *E. coli* infection with MOI 10 and relative average intensity analysis (n=3, each consisting of 4 random view fields). (D-F) Corresponding expression pattern of RNase7 in TERT-NHUC at mRNA and protein level. *E. coli* survival after incubation for (G) 6 h in conditioned media and (H) 30 min in the cell extract of TERT-NHUC uroepithelial cells (n=3). (I) Intracellular survival of *E. coli* after 2 h post gentamicin treatment and compared to initial inoculum (n=4). Survival assays were performed in duplicates or triplicates. Metformin (Met, 4mM) was treated for either 24 h or 36 h for mRNA or protein expression, bacterial survival assay respectively. Data are presented as mean with SEM. Significance levels *P<0.05, **P<0.01, ***P<0.001.

**Supplementary Movies**

**Movie 1. Time –lapse imaging of calcium dynamics upon metformin cell stimulation.** Uroepithelial cells, TERT-NHUC loaded with calcium indicator Fluo-4 AM (green) and treated with metformin followed by ionomycin at mentioned time points. Movie speed 20 frames per second.

**Movie 2. Time –lapse imaging of calcium dynamics upon AITC cell stimulation.** Uroepithelial cells, TERT-NHUC loaded with calcium indicator Fluo-4 AM (green) and treated with TRPA1 activator AITC followed by ionomycin at designated time points. Movie speed 20 frames per second.

**Movie 3. Time –lapse imaging of calcium dynamics upon AITC stimulation of metformin pre-treated cells.** Uroepithelial cells, TERT-NHUC pretreated with metformin for 1 h, loaded with calcium indicator Fluo-4 AM (green) and stimulated with TRPA1 activator AITC followed by ionomycin at designated time points. Movie speed 20 frames per second.

**Movie 4. Time –lapse imaging of calcium dynamics upon AITC stimulation to A96 pre-treated cells.** Uroepithelial cells, TERT-NHUC pretreated with TRPA1 inhibitor A-967079 (A96) for 1 h, loaded with calcium indicator Fluo-4 AM (green) and stimulated with TRPA1 activator (AITC) followed by ionomycin at the marked time points. Movie speed 20 frames per second.
